# Nasopharyngeal Endoscopic Resection Extended to the Internal Carotid Artery (Type 4): Technical Notes, Indications, and Outcomes

**DOI:** 10.1002/hed.70008

**Published:** 2025-08-20

**Authors:** Francesco Boaria, Alessandro Vinciguerra, Stefano Taboni, Martina Conti, Paola Costantino, Vittorio Rampinelli, Marco Valentini, Francesca Gennarini, Benjamin Verillaud, Elisabetta Zanoletti, Davide Mattavelli, Alberto Schreiber, Mario Turri‐Zanoni, Alberto Daniele Arosio, Piergiorgio Gaudioso, Paolo Battaglia, Maurizio Bignami, Paolo Castelnuovo, Philippe Herman, Piero Nicolai, Marco Ferrari

**Affiliations:** ^1^ Otorhinolaryngology and Skull Base Center, AP‐HP, Hospital Lariboisière Paris France; ^2^ Section of Otorhinolaryngology‐Head and Neck Surgery, Department of Neuroscience University of Padua Padua Italy; ^3^ Unit of Otorhinolaryngology‐Head and Neck Surgery University of Brescia Brescia Italy; ^4^ Division of Otorhinolaryngology, Department of Biotechnology and Life Sciences University of Insubria Varese Italy; ^5^ Université Paris Cité Paris France; ^6^ Inserm U1141 Paris France; ^7^ Unit of Otorhinolaryngology – Head and Neck Surgery Azienda Ospedale‐Università Padova Padova Italy

## Abstract

**Background:**

Endoscopic surgical approaches for nasopharyngeal malignancies, including recurrent nasopharyngeal carcinoma (rNPC) and nasopharyngeal salivary gland tumors (NSGT), have seen significant development over the last decades. Our groups have expanded the classification of nasopharyngeal endoscopic resections (NER) from type 1 to 3 to incorporate internal carotid artery (ICA)‐including ablations. The aim of this work is to describe the surgical technique, indications, and preliminary oncological outcomes of NER extended to ICA, proposing it as type 4 NER.

**Methodology:**

Consecutive patients affected by nasopharyngeal malignancies and treated with NER type 4 were included. Data on patient‐, disease‐, and treatment‐related factors were collected. Adverse events were classified from G1 to G5 according to the Common Terminology Criteria for Adverse Events. Preliminary survival outcomes were measured. A detailed description of the surgical steps of NER type 4 was reported.

**Results:**

A total of 13 patients were included: 7 NPC and 6 non‐NPC, of which 4 were primary adenoid cystic carcinoma (ACC), 1 was recurrent ACC, and 1 was polymorphous adenocarcinoma. After a median follow‐up of 34.7 months, 5 patients had a local recurrence during follow‐up after a median of 17.3 months, one of which died at 10.3 months after surgery. The cumulative ≥ G3 adverse event rate was 61.5%. No major complications directly associated with ICA occlusion were reported.

**Conclusions:**

NER type 4 is a potential treatment alternative to (re)irradiation in highly selected patients with nasopharyngeal malignancies involving/abutting the ICA. The results reported should be considered preliminary; a novel description of the technique is provided.

## 1 Introduction

Nasopharyngeal carcinoma (NPC) is the most frequent malignant tumor of the nasopharynx, whereas nasopharyngeal salivary gland tumors (NSGT), including adenoid cystic carcinoma (ACC), are infrequent [1, 2, 3, 4]. The primary treatment for NPC is radiotherapy (RT) or chemo‐radiotherapy (CRT), preceded by induction chemotherapy in selected cases, with surgery reserved for persistent or recurrent NPC (rNPC) [5, 6, 7, 8, 9, 10]. Studies examining NSGT are scarce, but upfront surgery has been shown to improve survival outcomes. Surgery with adjuvant RT is considered the standard of care for ACC and most NSGT [1, 11, 12, 13, 14].

Nasopharyngeal oncologic surgery can be performed through both open anterior and lateral approaches [15, 16, 17]. In recent years, endoscopic resections have shown to be a valuable alternative by avoiding external incisions and leading to a shorter hospital stay, a more favorable morbidity profile, and non‐inferior overall survival (OS) [15, 17, 18, 19, 20, 21, 22]. To provide a formal and reproducible description of the endoscopic technique, in 2010 Castelnuovo et al. described a classification for nasopharyngeal endoscopic resections (NER), with types 1 and 2 NER entailing midline resection of the posterosuperior part of the nasopharynx (without and with removal of the sphenoidal floor and anterior wall, respectively), and type 3 extending laterally to include the Eustachian tube and parapharyngeal space in the resected tissues [23]. Traditionally, the presence of internal carotid artery (ICA) involvement has been considered a criterion to define unresectable a head and neck cancer and thus a contraindication to NER and any type of surgery [24]. More recently, several studies have challenged the concept that ICA involvement/abutment is a contraindication to oncologic ablation [25, 26, 27].

Other authors have described endoscopic nasopharyngectomy procedures that can include ICA resection when the artery is involved by the tumor, proposing new classifications based on 4 types of nasopharyngectomies [22, 28, 29]. Similarly, our groups have expanded the classification of NER procedures to include ICA‐including ablations.

Herein, we aim to describe the surgical technique, indications, and preliminary oncological outcomes of NER extended to ICA, proposing it as type 4 NER according to the modified Castelnuovo classification, as recently reported [30].

## 2 Materials and Methods

A retrospective analysis was performed on consecutive patients who underwent NER including ICA resection in 2013–2024 in the following four centers: Azienda Ospedale Università Padova (Padova, Italy), Ospedale di Circolo e Fondazione Macchi in Varese (Varese, Italy), ASST Spedali Civili di Brescia (Brescia, Italy), and Hôpital Lariboisière (Paris, France).

Data on patient‐, disease‐, and treatment‐related factors were collected. Adverse events were classified from G1 to G5 according to the Common Terminology Criteria for Adverse Events (CTCAE), version 5.0 [31].

The study was conducted according to the ethical standards established in the Declaration of Helsinki, revised in 2011, and approved by the ethics committee of the coordinating center (“Comitato Etico per la Sperimentazione Clinica della Provincia di Padova”; protocol n. 0031745). Data were pseudonymized by the local principal investigator and handled in a fully anonymized fashion at the time of analysis. All patients consented to the use of anonymized clinical data for research purposes.

### 2.1 Surgical Technique

All patients underwent transarterial angiography of the carotid systems and ICA temporary balloon occlusion (TBO) test. Patients with a positive occlusion test for brain ischemia underwent extracranial‐to‐intracranial (EC‐IC) bypass surgery with a radial artery graft connecting the external carotid artery system and middle cerebral artery ipsilateral to the abutted/encased ICA, which was occluded at the same time. Patients with negative TBO underwent ICA coil‐occlusion at least 48 h prior to oncologic surgery.

The ablative phase of surgery started with a medial maxillectomy, aimed to gain adequate surgical exposure: the medial maxillary wall was removed up to the nasal floor inferiorly, palatine bone posteriorly, orbital floor superiorly, and nasolacrimal duct or anterior maxillary wall anteriorly, depending on the lateral extension of the tumor [32, 33, 34].

The surgical approach then proceeded with a posterior septectomy, ethmoidectomy, trans‐rostral sphenoidotomy, resection of the posterior wall of the maxillary sinus, and exposure of the pterygopalatine fossa. The descending palatine, palatovaginal, and vidian bundles were identified, cauterized, and divided. If not included in the targeted tissue to ablate, the pterygopalatine fossa content was left enveloped in the periosteum and displaced laterally. A transpterygoid approach was performed by drilling at least the root and medial pterygoid plate, using the vidian canal as a landmark for the anterior genu of the ICA. When lateral extension into the upper parapharyngeal space and/or partial resection of the lateral pterygoid muscle (LPM) was needed, the lateral pterygoid plate was also removed. The LPM and medial pterygoid muscles (MPM) were identified alongside the tensor (TVPM) and levator veli palatini muscles (LVPM), the latter running parallel to the Eustachian tube. The bony floor of the sphenoid sinuses was drilled out to the coronal plane at the level of the paraclival ICA. A full‐thickness incision was made in the nasopharyngeal vault and posterior wall, including mucosa, submucosal tissue, superior constrictor muscle, and prevertebral fascia and muscles (when needed): the superior limit corresponded to the previously drilled sphenoid floor; the inferior limit was outlined at the passage between the naso‐ and oro‐pharynx; on the lateral aspect, the Eustachian tube and surrounding soft tissues were included in the resection.

Exposure and resection of the ICA was tailored according to tumor extension. The carotid canal was identified and drilled in its paraclival and/or petrous portions to expose the artery. The parapharyngeal ICA was identified by dissecting the adipose space between the TVPM and MPM [35]. A Doppler probe was not used because of ICA occlusion. ICA resection was achieved by performing proximal and distal cuts at the level of the parapharyngeal and paraclival tracts, respectively, and dissecting the vessel off the surrounding tissues. When resection of the parapharyngeal ICA had to be extended close to the bifurcation, a transcervical approach to the parapharyngeal space was performed to ligate and divide the ICA at its origin.

**FIGURE 1:**
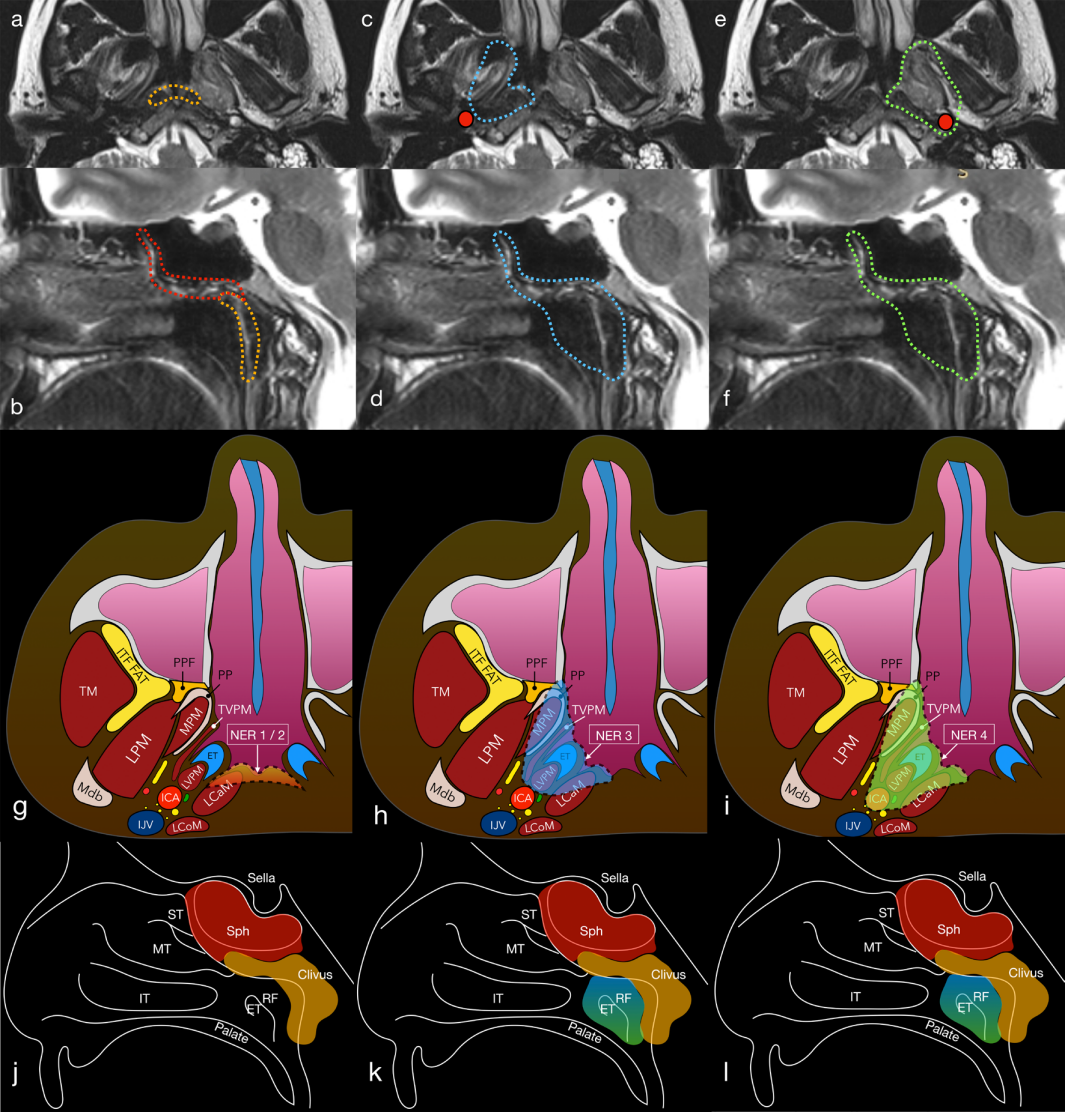
Surgical resection limits of the different types of NERs are highlighted by the dotted lines on magnetic resonance images of a patient with a left‐sided nasopharyngeal malignancy abutting the ipsilateral internal carotid artery (a–f). NER type 1 and 2 surgical limits seen on an axial plane (a), corresponding laterally to the Rosenmüller fossa (lateral recess of the nasopharynx), preserving the Eustachian tube; NER type 1 (yellow) and 2 (yellow + red) surgical limits seen on a sagittal plane (b), corresponding inferiorly to the naso‐oropharyngeal passage and superiorly to the floor of the sphenoid sinus, in case of NER 1, or including the floor and anterior wall of the sphenoid sinuses in case of NER 2; NER type 3 surgical limits seen on axial (c) and sagittal (d) planes, corresponding superiorly and inferiorly to those of the NER type 2 but more laterally extended to include the Eustachian tube; the ICA is highlighted with a red circle; type 4 NER seen on axial (e) and sagittal (f) planes. The ICA, highlighted with a red circle, is included in the resection which is further laterally extended compared with NER type 3. The surgical limits relative to relevant anatomical structures can be seen in images g to l, with yellow and red areas corresponding to NER 1 and 2 respectively, the blue area corresponding to NER type 3, and the green area to NER type 4. ET, Eustachian tube; ICA, internal carotid artery; IJV, internal jugular vein; IT, inferior turbinate; ITF, infratemporal fossa; LCaM, longus capitis muscle; LCoM, longus colli muscle; LPM, lateral pterygoid muscle; LVPM, levator veli palatini muscle; Mdb, mandible; MPM, medial pterygoid muscle; MT, middle turbinate; PP, pterygoid process; PPF, pterygopalatine fossa; RF, Rosenmüller fossa; Sph, sphenoid sinus; ST, superior turbinate; TM, temporalis muscle; TVPM, tensor veli palatini muscle. [Color figure can be viewed at wileyonlinelibrary.com]

**FIGURE 2:**
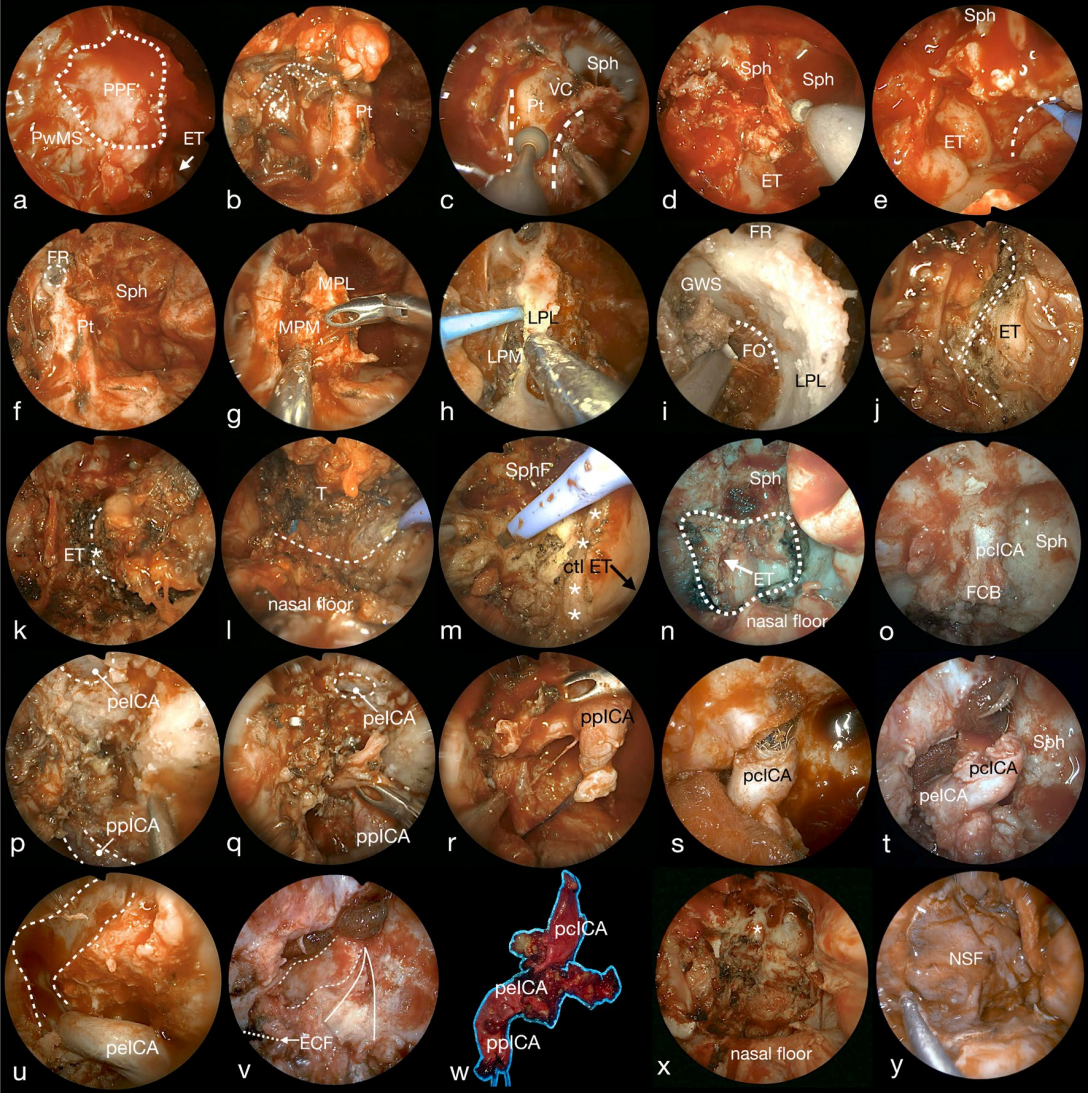
Panel displaying the main surgical steps of type 4 NER, right side. After medial maxillectomy is completed, the posterior wall of the maxillary sinus (PwMS) is visible and, after its removal, the periosteum of the pterygopalatine fossa (PPF) is exposed (a); during PPF dissection, the maxillary artery is identified, clipped and divided (dotted line) and the pterygoid process is exposed (Pt) (b); the vidian canal (VC) is visible at the base of the pterygoid process (dashed lines), which is then drilled (c) alongside with the bony floor of the sphenoid body (d); in the posterior wall the nasopharynx the dashed vertical line indicates the medial (i.e., contralateral) limit of the resection (e); after drilling the pterygoid process at the level of the vidian canal (f), the medial pterygoid lamina (MPL) is removed, exposing the medial pterygoid muscle (MPM) (g), and the lateral pterygoid muscle (LPM) is identified lateral to the lateral pterygoid lamina (LPL) (h); the dissection is continued laterally along LPL to expose the greater wing of the sphenoid (GWS), identifying the mandibular branch of the trigeminal nerve passing through the foramen ovale (FO) (i); dissection is then continued in the upper parapharyngeal space to identify the Eustachian tube (ET), which is sectioned close to the bony‐cartilaginous passage, defining the deep lateral limit of the resection (the asterisk indicates the lumen of ET) (j, k); the inferior limit of the resection is outlined at the naso‐oropharyngeal passage (dashed line) with monopolar electrocautery (l), and the specimen is dissected off the deep limit at the level of the prevertebral fascia/muscles (asterisk line = medial resection limit, ctl ET = contralateral ET, SphF = drilled sphenoid floor) (m); in the subsequent image (n) all the resection limits can be seen at once; exposure of the paraclival ICA (pcICA) and fibrocartilago basalis (FCB) is achieved by drilling the carotid sulcus (o); the petrous ICA (peICA) is exposed after drilling the carotid bony canal (dashed line, superior aspect of the image) and the parapharyngeal ICA (ppICA) is visible being pulled medially after being ligated and sectioned, in this case via a transcervical approach (inferior aspect of the image) (p–r); next the distal carotid is sectioned at the pcICA superior limit, with exposure of the coils used for vessel occlusion (s, t); the exposed carotid bony canal (dashed lines) can be visualized when the ICA is pulled infero‐medially (u) and after pcICA and peICA resection, with the inferior limit being at the level of the external carotid foramen (ECF); the continuous “reversed V‐shaped” line marks the petroclival junction and its soft tissue content (v); the resected ICA is then visible with its three portions (w); the entire surgical defect is visible in the subsequent image, with the asterisk indicating the distal carotid stump (x); in this case, reconstruction was performed with ileo‐tibial tract, right temporoparietal fascia flap, and left nasoseptal flap (NSF) (y). FR, foramen rotundum; Sph, sphenoid sinus. [Color figure can be viewed at wileyonlinelibrary.com]

**FIGURE 3:**
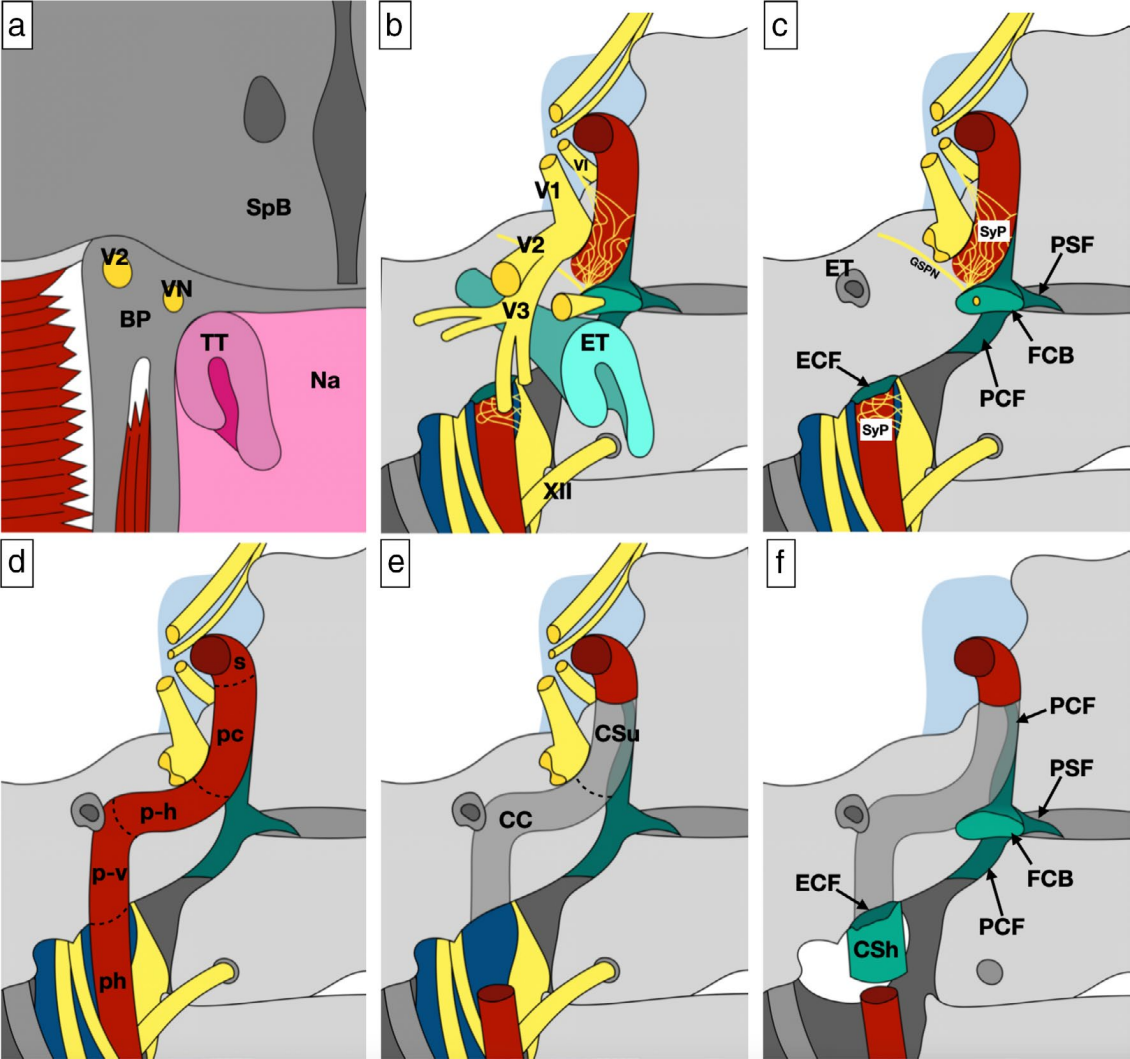
Panel summarizing the layer‐by‐layer relevant surgical anatomy of a type 4 nasopharyngeal endoscopic resection. (a) Scheme depicting the sphenoid body (SpB), nasopharynx (Na), and torus tubarius (TT); on the lateral aspect, the base of the pterygoid process (BP), with vidian (VN) and maxillary (V2) nerves, alongside with pterygoid plates and muscles, are shown. (b) Scheme showing the complex network of structures of this anatomical area, including the ophthalmic (V1), maxillary (V2), and mandibular (V3) branches of the trigeminal nerve and Eustachian tube (ET). (c) Scheme showing the relevant anatomy after removing the cartilaginous Eustachian tube alongside with the maxillary and mandibular branches of the trigeminal nerve; the greater superficial petrosal nerve (GSPN) crosses the petrous apex cranial surface to join the sympathetic internal carotid plexus (SyP) to form the vidian nerve into the anterior foramen lacerum; the latter houses the fibrocartilago basalis (FCB), which is tightly attached to the Eustachian tube and directly connected with other fibrocartilaginous areas including the petroclival (PCF) and petrosphenoidal (PSF) fissures. (d) Scheme showing the targetable tracts of the internal carotid artery, namely the parapharyngeal (ph), petrous vertical (p‐v), petrous horizontal (p‐h), and paraclival (pc) segments. (e) Scheme displaying the bony boundaries resulting after resecting the internal carotid artery, including the carotid canal (CC) and carotid sulcus (CSu), whereof the anterior aspect is removed to expose the paraclival tract of the internal carotid artery. (f) Scheme exemplifying critical areas to be checked after internal carotidectomy, since they represent potential routes of spread of cancers abutting the internal carotid artery; these are the petroclival fissure, petrosphenoidal fissure, fibrocartilago basalis, the fibroucartilaginous ring covering the external carotid foramen (ECF), and carotid sheath (CSh). VI, abducens nerve; XII, hypoglossal nerve; s, parasellar tract of the internal carotid artery. [Color figure can be viewed at wileyonlinelibrary.com]

After resecting the ICA, additional removal of the petrous apex, petroclival junction, jugular tuberculum, and occipital condyle was performed depending on oncologic needs. According to the principles of multi‐block resection [36, 37], all anatomical specimens were named and oriented to determine tumor extension and margin status. Details on the surgical limits of the different types of resections are displayed in Figure 1, while Figure 2 shows the relevant steps of the procedure. In Figure 3, relevant details on the surgical anatomy of type 4 NER are summarized. Video S1 shows the main surgical steps of a type 4 NER.

**FIGURE 4:**
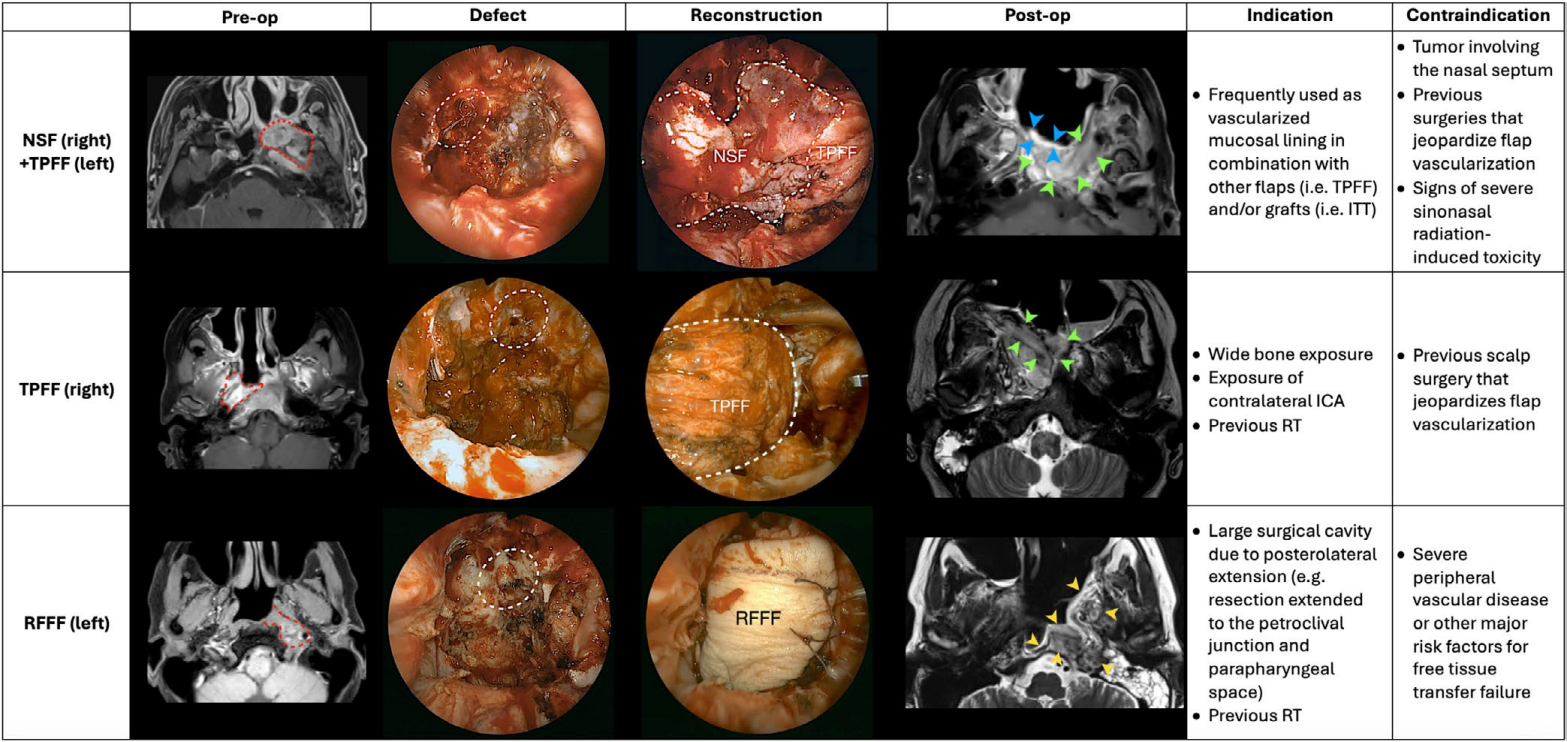
Panel displaying different reconstruction solutions and their indications and contraindications (NSF = nasoseptal flap; TPFF = temporoparietal fascia flap; RFFF = radial forearm free flap). In the first column, pre‐operative MRI images are reported. In the second and third columns, surgical defect and endoscopic intra‐operative images of the reconstruction are shown, respectively. In the fourth column, post‐operative MRI images are shown, where the NSF is marked by blue arrowheads, TPFF by green arrowheads, and RFFF by yellow arrowheads. In the first row, right NSF is used in combination with left TPFF after a left type 4 NER for a rNPC; coils from the paraclival tract of the resected left carotid artery are highlighted by the dashed line; the right NSF (dashed line) is visible superficial to the left TPFF. In the second row, right TPFF is used after a type 4 NER for a right ACC; coils from the paraclival tract of the resected right carotid artery are highlighted by the dashed line. In the third row, left RFFF transposed through a retropharyngeal corridor is used after a type 4 NER for a left rNPC; coils from the paraclival tract of the resected left carotid artery are highlighted by the dashed line. [Color figure can be viewed at wileyonlinelibrary.com]

Once resection was completed, reconstruction was carried out with different options depending on the extent of the defect and previous treatments, resorting to local, regional, or free flaps and autologous grafts. Figure 4 portrays the main different reconstructive solutions after the resection and their indications and contraindications.

## 3 Results

Thirteen patients who underwent type 4 NER were included in the present study, with a median follow‐up of 34.7 months. The median age at tumor diagnosis was 45.0 years (range: 23–65). Seven patients presented with rNPC, 4 with primary ACC, 1 with recurrent ACC, and 1 with polymorphous adenocarcinoma. Primary treatment in patients with local recurrence had included CRT in seven (one of which was re‐irradiated with proton beam RT with concomitant chemotherapy prior to surgery) and RT in 1. At definitive pathology, the margins were microscopically involved in 7 cases (5 ACC and 2 NPC). Carotid wall (i.e., tunica media) invasion was observed in 4 cases, and all the remainder but 1 patient (#13) showed infiltration of connective areas/tissues tightly attached to the vessel wall (i.e., foramen lacerum, petroclival junction soft tissues, and other adventitial tissues).

Patient‐, disease‐, and treatment‐related data are summarized in Tables 1 and 2. Two patients needed an EC‐IC bypass prior to surgery on the nasopharynx. Selection of candidates for EC‐IC bypass surgery was strict and based on previously reported criteria [25]. Patients with a positive TBO test were properly counseled on therapeutic strategies other than surgery, and a shared decision considering risks and benefits was made.

**TABLE 1:** Summary of patient‐, disease‐, and treatment‐related factors.

Clinical and treatment characteristics	*N*	Notes
Age (years), median (range)	45.0 (23–65)	
Gender M:F	6:7	
Follow‐up (months), median (range)	34.7 (7.5–81.1)	
Histology		
NPC	7	
non‐NPC	6	
ACC	5	
Salivary non‐ACC	1	
Patients treated with up‐front NER	5	4 ACC + 1 polymorphous adenocarcinoma
Patients treated before NER	8	
CRT	6	5 NPC + 1 ACC
RT	1	NPC
CRT + re‐(chemo)radiation	1	NPC
cT at primary treatment (any)		
cT1	1	
cT2	2	
cT3	4	
cT4	5	
Unknown	1	
cT at NER‐treated lesion diagnosis		
cT1	1	
cT2	2	
cT3	3	
cT4	7	
pT		
1	1	
2	2	
3	2	
4	8	
Pathologic degree of ICA involvement		
ICA wall	4	
FCB/PCJ	8	
Other pericarotid tissues	11	
Reconstruction		
Local flap	1	1 NSF
Regional flap	10	7 TPFF (± NSF and/or BFP); 3 BFP + ITT (±NSF (2) or MTF+ITF (1))
Free flap	2	1 ALTF^a^, 1 RFFF^b^
Length of hospitalization (days), median	10.0	Range: 5–30 days
Patients who received adjuvant treatment		
Type of treatment	6	1 NPC, 5 non‐NPC
CT	1	NPC
RT	5	5 non‐NPC

Abbreviations: ACC, adenoid cystic carcinoma; ALTF, antero‐lateral thigh flap; BFP, Bichat fat pad; CRT, chemo‐radiotherapy; CT, chemotherapy; FCB, fibrocartilago basalis; ICA, internal carotid artery; ITF, inferior turbinate flap; ITT, ileo‐tibial tract; MTF, middle turbinate flap; NSF, nasoseptal flap; PCJ, petroclival junction; RFFF, radial forearm free flap.

^a^ Pedicle positioned through the parapharyngeal corridor [38].

^b^ Pedicle positioned through the prevertebral corridor [38].

**TABLE 2:** Patients included in the present series.

Patient	Age (years), gender	Histology	Pre‐NER treatment	cT at primary treatment	cT at type 4 NER ‐treated lesion diagnosis	pT	ICA wall (tunica media) invasion	FCB/PCJ invasion	Other pericarotid tissue invasion	Margin status	Reconstruction	Length of hospitalization (days)	Adjuvant treatment	Adverse event(s) ≥ G3	Months of follow‐up (status at latest contact)
#1	48, M	dNK‐NPC	RT	1	4	3	No	Yes (FCB)	Yes (VCC, PPCS)	R0	ALT	30	None	Yes (synechiae)	30.1 (AWD)
#2	45, F	uNK‐NPC	CRT	2	2	2	No	No	Yes (PPCS)	R0	RFFF	10	None	Yes (ORN)	42.0 (NED)
#3	65, M	dNK‐NPC	CRT	3	3	4	No	No	Yes (HCC)	R1	TPFF + NSF	12	None	Yes (otitis media)	35.4 (AWD)
#4	42, M	uNK‐NPC	CRT + re‐chemo‐radiation with PBRT	3	3	3	No	Yes (FCB)	Yes (CSu)	R0	TPFF	25	None	No	47.5 (AWD)
#5	53, M	uNK‐NPC	CRT	3	2	2	No	Yes (both)	No	R0	TPFF	5	CT	Yes (ORN)	81.1 (NED)
#6	24, F	ACC	None	4	4	4	No	Yes (both)	Yes (CSu, HCC, VCC)	R1	ITT + NSF + BFP	10	PT	Yes (oculomotor nerve paresis)	40.6 (NED)
#7	54, F	Salivary gland polymorphous adenocarcinoma	None	3	3	4	Yes	Yes (FCB)	Yes (HCC)	R0	BFP + ITT + autologous fat + NSF	7	RT	No	62.8 (NED)
#8	43, F	ACC	CRT	2	4	4	Yes	Yes (FCB)	Yes (HCC, VCC, PPCS)	R1	NSF	7	None	No	10.3 (DOD)
#9	23, F	ACC	None	4	4	4	Yes	No	Yes (CSu, HCC, VCC)	R1	ITT + TPFF + NSF	10	RT	Yes (otitis media)	15.7 (NED)
#10	43, M	dNK‐NPC	CRT	Unknown	4	4	No	No	Yes (PePS, CSu)	R1	MTF + ITF + BFP + ITT	10	None	Yes (severe neuralgia)	34.7 (NED)^a^
#11	36, F	ACC	None	4	4	4	No	Yes (both)	Yes (CSu, HCC, VCC)	R1	BFP + NSF + TPFF	14	PT	Yes (palatal fistula)	7.5 (NED)
#12	62, F	ACC	None	4	4	4	Yes	Yes (both)	Yes (CSu, HCC, PePS, PPCS, VCC)	R1	BFP + NSF + TPFF	6	PT	No	8.4 (NED)
#13	46, M	uNK‐NPC	CRT	4	1	1	No	No	No	R0	NSF + TPFF	11	None	No	10.2 (NED)

Abbreviations: ACC, adenoid cystic carcinoma; ALTF, anterolateral thigh flap; AWD, alive with disease; BFP, Bichat fat pad; CRT, chemo‐radiotherapy; CSu, carotid sulcus; CT, chemotherapy; dNK‐NPC, differentiated non‐keratinizing NPC; DOD, dead of disease; FCB, fibrocartilago basalis; FF, free flap; HCC, horizontal carotid canal; ITF, inferior turbinate flap; ITT, ileo‐tibial tract; LF, local flap; MTF, middle turbinate flap; NED, no evidence of disease; NSF, nasoseptal flap; ORN, osteoradionecrosis; PBRT, proton beam radiotherapy; PCJ, petroclival junction; PePS, periosteum of the inferior parasellar area (Meckel's cave and superior orbital fissure); PPCS, parapharyngeal carotid sheath; PT, proton therapy; RF, regional flap; RFFF, radial forearm free flap; RT, radiotherapy (photons); uNK‐NPC, undifferentiated non keratinizing NPC; VCC, vertical carotid canal.

^a^ The patient had a local recurrence into the orbital apex and dura of the middle cranial fossa 17 months after surgery, which was treated with definitive re‐RT with complete response.

Six patients received adjuvant treatment after type 4 NER: 1 patient received adjuvant chemotherapy for a rNPC and 5 patients received postoperative RT (3 ACC treated with proton RT, and 1 ACC and 1 salivary polymorphous adenocarcinoma treated with photon‐based intensity‐modulated RT). None of the patients who received adjuvant RT had undergone irradiation before surgery.

The most frequently reported complication was otitis media with effusion (7 patients). Osteoradionecrosis was reported in 2 patients; since both cases required surgery, the complication was classified as G4. Two patients, who developed extraocular muscle paresis (patient #6: III cranial nerve palsy, classified as G3; patient #13: VI cranial nerve palsy, classified as G2), completely recovered muscle function within 3 months. One patient developed a palatal fistula, which was classified as G3 since a surgical treatment (primary closure) was needed. Thus, 4 cases account for the entirety of surgical site‐related grade ≥ G3 adverse events. Our results translate to an overall ≥ G3 adverse event rate of 61.5%. No major complications directly associated with ICA occlusion were reported. Details regarding the adverse events are listed in Table 3.

**TABLE 3:** Adverse events. “Worst adverse event” represents the highest grade of complication reported for each patient, regardless of the total number of complications reported.

Adverse events	*N*	Notes
Frequency of adverse events reported after NER, w/o adjuvant treatment (no. of patients who reported an adverse event of that grade)		
G1	4	– G1: headache, trigeminal nerve disorder (2 patients), epiphora, otitis media, Bernard‐Horner syndrome – G2: dysphagia, otitis media (4 patients), pulmonary embolism, respiratory infection, hypoacusia, wound dehiscence, oculomotor nerve disorder, abducens nerve paresis – G3: nasal synechiae, otitis media (2 patients), neuralgia, oculomotor nerve paresis – G4: ORN (2 patients)
G2	8	
G3	6	
G4	2	
G5	0	
Overall ≥ G3	8	
Worst adverse event		
None	0	G3 events reported as worst complication – Synechiae, 1 patient (required surgery) – Otitis media, 2 patients – Neuralgia, 1 patient – Palatal fistula, 1 patient (surgery for primary closure) – Oculomotor nerve paresis, 1 patient G4 events reported as worst complication – ORN, 2 patients
G1	1	
G2	2	
G3	6	
G4	2	
G5	0	

Abbreviation: ORN, osteoradionecrosis.

At the latest available follow‐up, 12 patients are alive, whereas 1 died at 10.3 months for a local recurrence diagnosed at 6.6 months after surgery. Five patients had a local recurrence during follow‐up after a median of 17.3 months. No regional or distant recurrences were reported. Outcomes at 2 years were as follows: 92.3% OS (95% CI: 75.4–100.0), 61.5% (95% CI: 22.8–93.6) local control rate, 61.5% (95% CI: 22.8–93.6) recurrence‐free survival, and 0% treatment‐related mortality.

## 4 Discussion

The present study focuses on type 4 NER, the most advanced and recently added procedure of the modified Castelnuovo classification of endoscopic nasopharyngectomies [30]. Other groups have already reported on the possibility of including ICA in the ablation during a nasopharyngectomy. However, their classifications of nasopharyngectomies are based on slightly different criteria than those used in our centers, such as the type 4 transnasal endoscopic nasopharyngectomy proposed by Liu et al. in 2021, which can also be applied to the resection of intracranial rNPC, or the type 4 resection described by Li et al. in 2022, which is primarily focused on the posterolateral extension but not necessarily always including ICA resection [28, 29]. Given the technical and clinical implications of resecting the ICA, it is our opinion that defining a type of NER specifically characterized by this surgical step is relevant, as previously suggested by Liu et al. [22].

The main finding of our study is that type 4 NER can be an option to treat selected patients with recurrent or persistent NPC and primary radioresistant nasopharyngeal malignancies with ICA involvement. Indeed, especially in the case of NPCs, it appears to be a valid alternative to re‐irradiation, avoiding the risk of life‐threatening complications (such as carotid blowout) in patients who have already received RT as primary treatment.

Data in the literature regarding salvage treatments for rNPC report a 42% rate of radiation‐induced toxicity (≥ G3) for IMRT, whereas the rate of surgical complications (≥ G3) is 5% for NER, showing that surgical treatment can provide lower morbidity compared to salvage IMRT [21]. Our data focus on NER extended to the ICA, which in 6 cases was associated with adjuvant therapy. In this respect, a comparison with other investigations in the literature is difficult to perform. For instance, a study on rNPC resection including ICA after EC‐IC bypass reported an overall complication rate of 25% (7 of 28 patients) [16]. In our study, we report an overall ≥ G3 adverse event rate of 61.5%. The difference might be related to the variability of the systems used to define adverse events. One of the most feared complications of ICA‐sparing treatments for head and neck cancers is carotid blowout, with a mean lethality of 50% in surgically treated and 76% in re‐irradiated patients, and an overall incidence up to 17% in re‐irradiated patients [25, 39, 40]. In our series, we report no major vascular events or mortality related to vascular complications.

Concerning survival outcomes, studies have shown the advantage of endoscopic approaches for rNPC (5‐year OS: 61%) compared to re‐IMRT (5‐year OS: 41%) [21, 22]. In the present study, which is centered on NER with ICA resection, the 2‐year OS estimate was 92.3%, highlighting the therapeutic potential of this aggressive surgical approach in highly selected cases of ICA abutment/enclosure. Of note, survival outcomes in patients affected by skull base malignancies depend on several factors, with histotype and biological behavior representing the most relevant. Therefore, the survival metrics reported in the present study are intended solely to demonstrate the adequacy of outcomes in a small, carefully selected cohort undergoing an advanced surgical procedure and should not be used as a benchmark for prognostic prediction.

When performing a type 4 NER, it is of utmost importance to ascertain sufficient brain hemodynamics with a TBO test. Only in case of test tolerance was the occlusion performed primarily [22, 25, 41, 42, 43]. Of note, two patients did not tolerate the TBO test and required EC‐IC with contextual ICA occlusion prior to oncologic surgery.

Regarding the extent of pathological carotid involvement, in our series ICA wall invasion was confirmed by pathology in four cases. Nevertheless, all the remaining cases but one showed infiltration of pericarotid connective tissues (i.e., thin connective areas separating the carotid muscular wall from adjacent bony or soft tissues) and/or neighboring connective areas such as fibrocartilago basalis (FCB) or petroclival junction (PCJ). Thus, in most cases, including those with tunica media‐free abutment, an ICA‐sparing resection would have been at high risk of intra‐ or post‐operative ICA blowout.

In addition, it is essential to consider tumor‐related (summarized in Table 4) and tumor‐unrelated contraindications of such an aggressive surgery. The patient's general condition must be thoroughly assessed during pre‐operative planning. General status and comorbidities play a key role in the ability to withstand such treatment and must be properly evaluated. This concerns not only the hemodynamic aspect related to ICA occlusion and resection, but also possible intra‐ and post‐operative complications ranging from potentially severe early adverse events to long‐term complications affecting quality of life. This goes along with the patient's motivation to undergo this type of treatment, considering that even a young and fit patient might not be eager to agree to the surgical option.

**TABLE 4:** Indications and contraindications of type 4 NER.

cT category	ICA tract potentially involved	Indication for NER type IV	Contraindication to surgery
T2	Parapharyngeal	Extension into the upper parapharyngeal space with limited involvement or abutment (i.e., very close relationship, contact) of the parapharyngeal tract of the ICA	—
T3	Parapharyngeal, petrous, paraclival	Extension to the sphenoid floor, clivus, and anterior arch of the atlas, with limited involvement or abutment (i.e., very close relationship, contact) of the anterior genu of the ICA and limited involvement of the adjacent tracts Limited extension to the carotid canal and its bony boundaries, petroclival junction, and pterygoid process with involvement or abutment (i.e., very close relationship, contact) of the horizontal petrous tract of the ICA	Bicortical extension through the clivus and/or anterior arch of the atlas Complete encasement of the paraclival tract of the ICA Complete encasement of the vertical petrous tract of the ICA
T4	Any	Extension into the parapharyngeal/masticator space lateral to the parasagittal plane passing through the lateral pterygoid process, with involvement or abutment (i.e., very close relationship, contact) of the ICA *Limited involvement of the extracranial tract of the XII c.n. adjacent to the occipital condyle, with involvement or abutment (i.e., very close relationship, contact) of the ICA	As per T3 Intracranial extension Hypopharyngeal involvement Orbital involvement Parotid gland involvement C.n. involvement (except for selected cases of XII* and V2/V3 c.n. extracranial/foraminal involvement)

On the other hand, the likelihood of achieving a complete resection must be properly weighed considering tumor extension to critical structures other than the ICA. In the case of bone involvement, it is more difficult to ensure clear bone resection margins compared to soft tissues. In particular, it has been shown that the chance of positive bone margins is even higher when the tumor invades both cortexes of the clivus or the lateral wall of the sphenoid sinus [27]. Similarly, extensive resection of the lateral wall of the sphenoid sinus is limited by the presence of critical structures other than the ICA, such as the optic nerve or cranial nerves III, IV, and VI [27]. Regarding the limitations related to ICA involvement, a type 4 NER may be envisaged when the tumor invades/abuts the anterior genu, the petrous portion, or, to a limited extent, the paraclival or parapharyngeal portions. Extensive involvement of these two vertical portions of the ICA would render the disease unsuitable for an endoscopic resection.

Finally, it is essential to emphasize the importance of proper reconstruction after resection, which is essential to facilitate healing and minimize complications. It has been suggested that reconstruction with a vascularized flap has a protective effect on the development of ≥ G3 adverse events, ensuring an adequate blood supply to previously irradiated tissues that have impaired microcirculation [9]. The choice of reconstructive strategy depends on several factors, including the depth and width of the defect, perfusion of local tissues, and need for transcervical access to the carotid bifurcation. In a study by Chan et al., a historical cohort (121 patients treated between 1990 and 2001) was compared with a more recent cohort (217 patients treated between 2002 and 2012) to evaluate morbidity following maxillary swing nasopharyngectomy for rNPC [44]. The authors observed a reduction in ICA blowout rates (from three cases in the earlier cohort to none in the later cohort) after the more extensive adoption of vascularized flap‐based coverage of the ICA. These findings underscore the importance of appropriate reconstruction techniques in preventing life‐threatening complications and support the continued relevance of ICA‐sparing open surgical approaches in selected cases of skull base malignancies abutting the ICA.

The limits of this study mainly reside in the small number of cases, short follow‐up, and heterogeneity of histologies included. This latter element is of notable importance since the study includes both NPCs and NSGTs, such as ACC and polymorphous adenocarcinoma. The variety of histologies might impact outcomes such as the rate of positive margins (5 of 7 R1 surgeries were represented by ACC, which is almost invariably associated with positive margins when involving the skull base and adjacent areas), as well as OS, which is generally more favorable in ACC compared to NPC, and the morbidity rate, which also depends upon suitability for chemo‐ and/or radio‐therapy prior to and/or after surgery. The high rate of R1 resections may raise questions about the appropriateness of non‐radical surgery for the ACC cases included in this study. However, our group adheres to the principle that optimal outcomes in patients with ACC of the ventral skull base are achieved through gross total resection (which rarely results in negative margins) followed by adjuvant radiotherapy. Therefore, ICA resection is considered justified if it allows for avoidance of R2 resection in these cases. However, the authors acknowledge that current evidence does not definitively establish the superiority of this approach for ACC and NSGT in general, [45] and that further research is needed.

## 5 Conclusions

This study describes a NER extended to the ICA that we propose to identify as type 4 NER. The technique is a potential treatment alternative to (re)irradiation in highly selected patients with nasopharyngeal malignancies involving/abutting the ICA. Although the results reported can be considered preliminary in relation to the small number of patients and relatively short follow‐up, a novel description of the technique is provided. Categorizing this approach as a surgical entity will help to collect more data and improve knowledge on outcomes and indications.

## Author Contributions

F. Boaria, A. Vinciguerra, S. Taboni, M. Conti, P. Costantino, D, V. Rampinelli, M. Valentini, F. Gennarini, B. Verillaud, D. Mattavelli, and M. Turri‐Zanoni made substantial contributions to conception, design and acquisition of data, drafted the article, revised it critically for important intellectual content, gave final approval of the version to be published, and agreed to be accountable for all aspects of the work in ensuring that questions related to the accuracy or integrity of any part of the work are appropriately investigated and resolved. P. Castelnuovo, M. Bignami, A, P. Battaglia, P. Nicolai, P. Herman and M. Ferrari made substantial contributions to the conception of the study, revised it critically for important intellectual content, gave final approval of the version to be published, and agreed to be accountable for all aspects of the work in ensuring that questions related to the accuracy or integrity of any part of the work are appropriately investigated and resolved.

## Disclosure

The authors have nothing to report.

## Conflicts of Interest

The authors declare no conflicts of interest.

## Supporting information


**Video S1:** Surgical video showing the steps of type 4 nasopharyngeal endoscopic resection in patient #12.

## Data Availability

The data that support the findings of this study are available on request from the corresponding author. The data are not publicly available due to privacy or ethical restrictions.
